# Hot Workability and Microstructure Evolution of Homogenized 2050 Al-Cu-Li Alloy during Hot Deformation

**DOI:** 10.3390/ma17174236

**Published:** 2024-08-27

**Authors:** Zhiyong Sheng, Yuanchun Huang, Yongxing Zhao, Rong Fu, Xucheng Wang, Xi Fan, Fan Wu

**Affiliations:** 1 Light Alloy Research Institute, Central South University, Changsha 410083, China; shengzy@innochina.ltd (Z.S.); ychuang@csu.edu.cn (Y.H.); 2 Hunan InnoChina Advanced Materials Co., Ltd., Yueyang 414021, China; zhaoyongxing@innochina.ltd (Y.Z.); csumingli@csu.edu.cn (X.F.); 15761647992@163.com (F.W.); 3 College of Mechanical and Electrical Engineering, Central South University, Changsha 410083, China; 4 State Key Laboratory of Precision Manufacturing for Extreme Service Performance, Changsha 410083, China; 5 School of Metallurgy and Environment, Central South University, Changsha 410083, China; 6 School of Mechanical and Automotive Engineering, Anhui Polytechnic University, Wuhu 241000, China; wangxuchengcsu@163.com

**Keywords:** 2050 Al-Cu-Li alloy, hot deformation behavior, constitutive modeling, processing map, microstructure, dynamic recrystallization

## Abstract

For this article, hot compression tests were carried out on homogenized 2050 Al-Cu-Li alloys under different deformation temperatures and strain rates, and an Arrhenius-type constitutive model with strain compensation was established to accurately describe the alloy flow behavior. Furthermore, thermal processing maps were created and the deformation mechanisms in different working regions were revealed by microstructural characterization. The results showed that most of the deformed grains orientated toward <101>//CD (CD: compression direction) during the hot compression process, and, together with some dynamic recovery (DRV), dynamic recrystallization (DRX) occurred. The appearance of large-scale DRX grains at low temperatures rather than in high-temperature conditions is related to the particle-stimulated nucleation mechanism, due to the dynamic precipitation that occurs during the deformation process. The hot-working diagrams with a true strain of 0.8 indicated that the high strain-rate regions C (300 °C–400 °C, 0.1–1 s^−1^) and D (440 °C–500 °C, 0.1–1 s^−1^) are unfavorable for the processing of 2050 Al-Li alloys, owing to the flow instability caused by local deformation banding, microcracks, and micro-voids. The optimum processing region was considered to be 430 °C–500 °C and 0.1 s^−1^–0.001 s^−1^, with a dissipation efficiency of more than 30%, dominated by DRV and DRX; the DRX mechanisms are DDRX and CDRX.

## 1. Introduction

Third-generation Al-Cu-Li aluminum alloys have the characteristics of low density, high strength, excellent corrosion resistance, and good fracture toughness. They are widely used for structural components in aerospace and other fields [1,2]. These structural components are frequently obtained from the cast ingots through heat treatment (e.g., homogenization, solution, and aging) and thermomechanical processing (e.g., forging, hot extrusion, and hot rolling) [3,4]. Regarding the Al-Cu-Li alloys, although the mechanical properties are greatly improved with the addition of various trace elements, the deterioration or reduction of hot workability limits their wide application. Furthermore, the deformation behavior of Al-Cu-Li alloys is influenced by various factors such as strain, deformation temperature, and strain rate, which subsequently contribute to the quality of the final components [5]. Therefore, it is necessary to investigate the deformation behavior and microstructure evolution of Al-Cu-Li alloys during plastic deformation and to determine the optimized parameters for thermo-mechanical processing.

During the hot deformation process, the variation of flow stress is determined by the combined interaction of dislocation accumulation and dislocation annihilation, which indicates the evolution of the microstructure indirectly; meanwhile, the level of flow stress suggests the deformation property of the alloy and determines the sizes of load and energy during deformation [6]. The quantitative relationship between flow stresses and machining parameters during the deformation of aluminum alloys can be expressed by a constitutive equation [7]. There are three main types of constructive models, namely, phenomenological, physically based, and artificial neural network (ANN)-based [8]. The available phenomenological models are based on empirical research, involve fewer material constants, and are easy to calibrate during their application. The Arrhenius-type phenomenological model is used extensively to depict the flow stress of Al alloy [9]. The machinability of alloys can be assessed by process maps based on dynamic material models (DMM), which reveal the microstructural evolution of plastic deformation under various conditions and optimize the process area to avoid unstable deformation [10]. The defined instability and power dissipation efficiency (*η*) are displayed in the processing diagram. Numerous studies have reported a relationship between η and softening mechanisms (i.e., dynamic recrystallization (DRX) and dynamic recovery (DRV), where DRX greatly affects the microstructure and properties of Al alloys [11,12]). At 0.2 ≤ *η* ≤ 0.3, DRV is the dominant softening mechanism; DRX dominates when 0.3 ≤ *η* ≤ 0.6, and superplastic deformation dominates when *η* ≥ 0.6 [13,14].

For Al-Cu-Li alloys with high stacking fault energy (SFE), DRV is typically more likely to occur due to the dissociation reactions that promote the cross-slip and creep of dislocations, resulting in a lower DRX driving force [11]. Based on the nucleation and growth paths of new grains without distortion, DRX can be classified into three types, i.e., discontinuous dynamic recrystallization (DDRX), continuous dynamic recrystallization (CDRX), and geometric dynamic recrystallization (GDRX) [15]. Among them, CDRX has no recrystallization nucleation and is obtained by the gradual rotation of sub-grains, which occurs mainly inside deformed grains or near the grain boundaries; DDRX is derived from nucleation by grain boundary bulging and growth via (sub)grain boundary migration, which occurs near the grain boundaries; GDRX grains are formed when the jagged grain boundaries of both thin and elongated deformed grains come into contact with each other, which can be observed under conditions of large strains [16]. The occurrence of DRX in Al-Cu-Li alloys has been reported in several studies; for example, Zhu et al. [17] found that the dynamic restoration mechanism of 2050 alloy under all compression conditions involves both dynamic recovery (DRV) and dynamic recrystallization (DRX). Zhang et al. [18] showed that the DRX mechanisms of 2195 Al alloy are DDRX and CDRX at medium and high temperatures, respectively, wherein GDRX dominates at high temperatures and in extensive deformation (480 °C—80% or 520 °C—60%). Wang et al. [19] reported that the coarse Al_2_CuLi phase in Al-Li alloys can promote the occurrence of DRX through particle-stimulated nucleation (PSN), which is conducive to good workability. For the DRX mechanism induced by PSN, the deformation process tends to form deformation regions around the secondary phase that are unconnected to the α-Al crystals, and the existence of an obvious misorientation gradient in these regions contributes to the formation of DRX [20].

Al-Li alloy is a precipitation-strengthened alloy with a complex type of secondary phase that is sensitive to thermal deformation conditions. Yang et al. [21] showed that in Al-Li alloys, the remelting of the secondary phase occurs at temperatures higher than 490 °C, resulting in flow instability. Zhong et al. [22] suggested that the coarse secondary phase of Al-Li alloys dissolves during deformation at 500 °C, while the fine secondary phase precipitates under strain-induced conditions. Zhu et al. [17] indicated that the precipitation of the secondary phase is noted in 2050 Al-Li alloy during deformation at 340 °C. The dissolution or precipitation behavior of the secondary phase is accelerated by the introduction of defects (e.g., vacancies, dislocations, stacking faults, etc.) during the deformation process, which affects strain hardening and dynamic softening (DRV and DRX), thus modifying the deformation behavior and the microstructure. For this paper, isothermal hot compression tests of 2050 Al-Cu-Li alloy were carried out at different deformation temperatures and strain rates. Based on the obtained stress–strain curves and on microstructure characterization by scanning electron microscope (SEM) and electron backscatter diffraction (EBSD), the deformation behavior and secondary phase and grain structure characteristics were analyzed. Furthermore, an Arrhenius-type constitutive model with the compensation of strain was developed to effectively predict the flow stresses, and processing diagrams were drawn to determine the plastic deformation parameters that are suitable for 2050 Al-Li alloy.

## 2. Experimental Procedures

**Figure 1:**
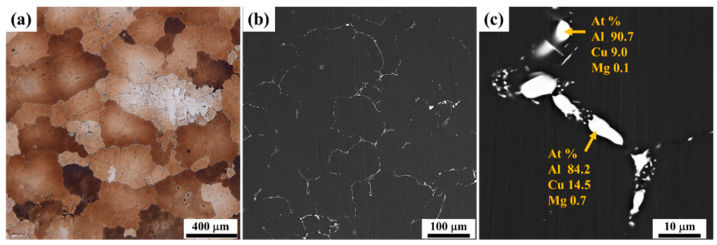
Microstructure of homogenized 2050 Al-Li alloy before hot compression: (**a**) OM image, (**b**,**c**) SEM image and EDS results.

The materials used for hot compression were 2050 Al-Cu-Li alloy ingots supplied by Hunan InnoChina Advanced Materials Co., Ltd. (Yueyang, China), homogenized at 465 °C for 8 h and at 500 °C for 24 h. The chemical composition was determined by inductively coupled plasma optical emission spectrometry (ICP–OES, PerkinElmer Avio500, Shelton, CT, USA), as shown in Table 1. The microstructure of 2050 Al-Li alloy treated with homogenization before hot compression deformation is shown in Figure 1. It can be seen that the grains of the homogenized 2050 Al-Li alloy exhibit an equiaxed shape with an average size of 275.0 μm (Figure 1a). There are coarse secondary phases near the grain boundaries but not inside the grains; these are mainly Al-Cu/Al-Cu-Li phases enriched with Mg elements, as indicated by the EDS results (Figure 1b,c).

**Figure 2:**
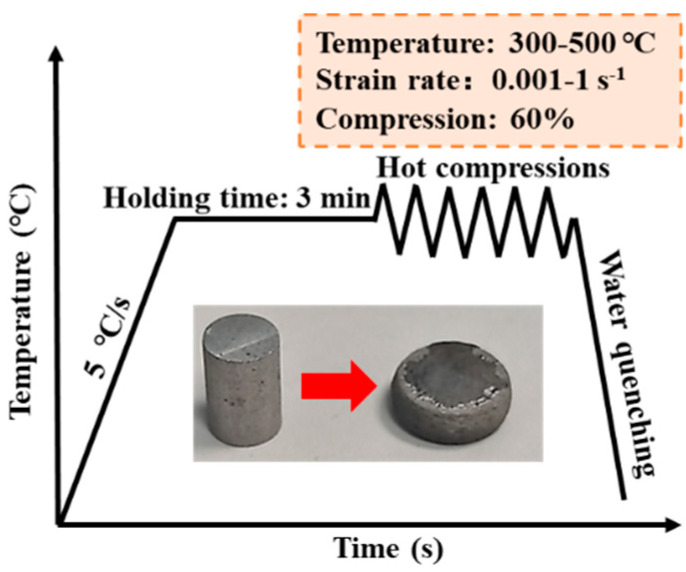
The schematic diagram of the hot compression experiments.

Using thermo-mechanical testing equipment (Gleeble-3810-GTC, DSI Co., Ltd., Dallas, TX, USA), standard cylindrical specimens of size Ф10 × 15 mm were taken from the center of homogeneous ingots of dimensions 120 × 120 × 40 mm and compressed. Graphite slices were placed at both ends of the compression specimens for lubrication. Stress-strain data were collected automatically for the period of compression testing and were not corrected for barreling effects, owing to the negligible level of barreling [23]. Generally, the heat distortion temperature should be higher than 0.4 Tm (Tm is the absolute temperature of the metal melting point) and lower than the over-burning temperature of Al-Li alloys, so the hot compression temperatures were selected to be 300 °C, 350 °C, 400 °C, 450 °C, and 500 °C, respectively [24,25]. In addition, the strain rates were 0.001 s^−1^, 0.01 s^−1^, 0.1 s^−1^, and 1 s^−1^, with 60% compression deformation (a true strain of 0.91). The specimens were heated to the compression temperature at a rate of 5 °C/s and maintained for 3 min to allow temperature uniformity. The short duration of the heating and holding stages before hot compression deformation has little effect on the secondary phase, and its microstructural characteristics are not significantly different from those of the homogenized specimens. After the compression experiments, the compression samples were water-cooled immediately to maintain the deformed microstructure. An illustration of the hot compression test is presented in Figure 2.

The deformed microstructures under different compression conditions were characterized using an SEM (FEI-Phenom, Thermo Fisher Scientific Inc., Bleiswijk, The Netherlands) with an energy disperse spectroscopy (EDS) and electron backscattered diffraction (EBSD) detector. The compressed specimens were divided into two halves in the direction parallel to the compression, and the profiled surfaces were mechanically polished for SEM examination. For EBSD tests, the electrolytic polishing was carried out in a mixture of 70% CH_3_OH and 30% HNO_3_ at a voltage of 25 V for 10 s to remove the influence of the stress layer on the observed microstructure. To process all the acquired EBSD data, the HKL Channel 5 software was used.

## 3. Results and Discussion

### 3.1. True Stress–Strain Curves

**Figure 3:**
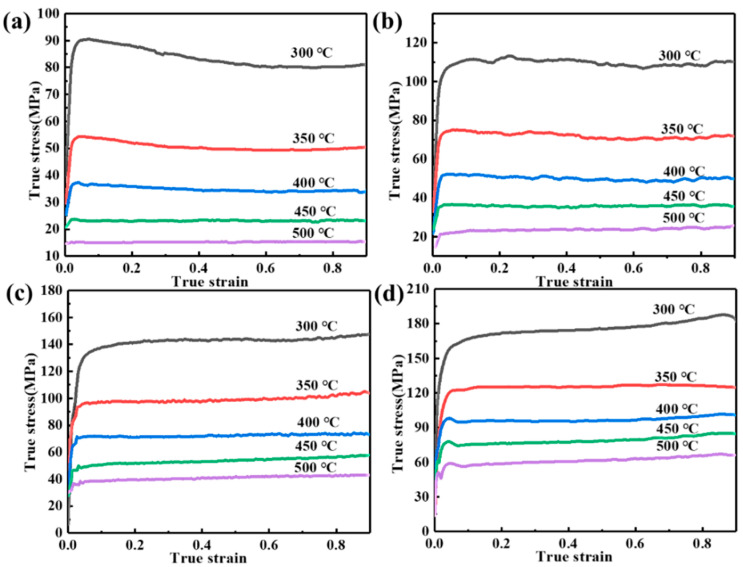
True stress–strain curves of samples under different deformation conditions: (**a**) 0.001 s^−1^; (**b**) 0.01 s^−1^; (**c**) 0.1 s^−1^; (**d**) 1 s^−1^.

Figure 3 shows the true stress–strain curves for different deformation conditions. The variation of flow stress during hot compression is the result of competing for work hardening and dynamic softening. In the initial stage of deformation (true strain of less than 0.05), the flow stress rises rapidly and the work-hardening rate (θ = dσ/dε, where σ and ε are the true stress and strain, respectively) is large; however, with the gradual increase in the true strain, the work-hardening rate decreases, and the growth of the flow stress decreases. The deformation consists of an elastic–plastic transition in the initial stage and a uniform plastic deformation in the subsequent stage [26]. The plastic deformation continuously generates dislocations, and the dislocation movement is hindered by the grain boundaries and mutual entanglement, which makes the dislocation density increase rapidly and, thus, produces a strengthening effect [27]. However, with continued deformation, the work-hardening rate decreases and reaches 0. The flow stress gradually increases and then reaches a steady state, which is related to the dynamic softening that occurs during deformation. The occurrence of DRV and DRX consumes some of the dislocations, resulting in lower dislocation density and leading to a reduction in the work-hardening effect. Once the dynamic softening rate is balanced with the work-hardening rate, the flow stress reaches a steady state. Moreover, flow stress behavior is extremely sensitive to deformation temperature and strain rate. The flow stress decreases with the increase in deformation temperature and increases with the increase in strain rate. This can be attributed to the fact that the higher activation energy provided by high temperatures facilitates atomic diffusion for dislocation annihilation and rearrangement, thus promoting DRV and DRX [28,29]. Nevertheless, at high strain rates, the duration of dynamic softening is shortened, limiting dislocation consumption and leading to higher flow stresses.

### 3.2. Constitutive Model with Compensation of Strain

The constitutive equation is a mathematical model that reflects the relationship between flow stresses and the hot deformation parameters of deformed alloys. Among the many constitutive models, the Arrhenius model is the one most commonly and effectively used to predict the hot deformation behavior of Al alloys, as shown below [30]:

(1) $$\dot{\varepsilon }=AF\left(\sigma \right)exp\left[-Q/(RT)\right]$$

where *F*(*σ*) is a function of the flow stress and can be expressed as:

(2) $$F\left(\sigma \right)=\left\{\begin{array}{c} \sigma^{n_{1}}\;(\alpha \sigma <0.8)\\ exp\left(\beta \sigma \right)\;\left(\alpha \sigma >1.2\right)\\ {\left[sinh\left(\alpha \sigma \right)\right]}^{n}\;for\;all\;\sigma \end{array}\right.$$

Among them, the first equation applies to low flow stresses (α*σ* < 0.8), the second equation applies to high flow stresses (α*σ* > 1.2), and the third equation is a sine-hyperbolic equation, which is suitable for a wide range of flow stresses. Here, $\dot{\varepsilon }$ (s^−1^) is the strain rate; *Q* (KJ/mol) is the hot activation energy; T (K) is the deformation temperature; R (8.314 J·mol^−1^·K^−1^) is the gas constant; *σ* (MPa) is the flow stress; A, n_1_, β, α (α = β/n_1_), and n are material constants.

Taking Equation (2) into Equation (1) and taking the logarithm of both ends of the equation, the following is obtained:

(3) $$\left\{\begin{array}{c} ln\sigma =\frac{1}{n_{1}}ln\dot{\varepsilon }+\frac{1}{n_{1}}\left(\frac{Q}{RT}-lnA\right)\;(\alpha \sigma <0.8)\\ \sigma =\frac{1}{\beta }ln\left(\dot{\varepsilon }\right)+\frac{1}{\beta }\left(\frac{Q}{RT}-lnA\right)\;\left(\alpha \sigma >1.2\right)\\ \ln\left[\sinh\left(\alpha \sigma \right)\right]=\frac{1}{n}ln\left(\dot{\varepsilon }\right)-\frac{1}{n}lnA+\frac{Q}{nRT}\;for\;all\;\sigma \end{array}\right.$$

**Figure 4:**
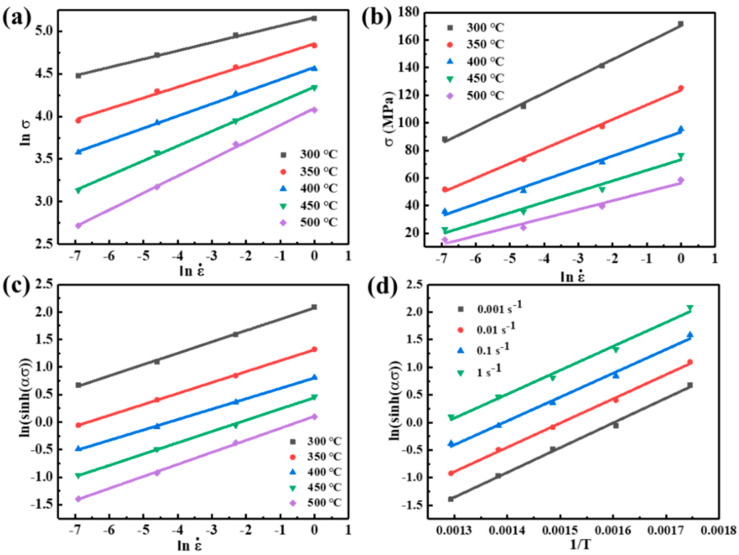
Linear fitting: (**a**) $ln\sigma -ln\dot{\varepsilon }$; (**b**) $\sigma -ln\dot{\varepsilon }$; (**c**) $\ln\left[\sinh\left(\alpha \sigma \right)\right]$ − $ln\left(\dot{\varepsilon }\right)$; (**d**) $\ln\left[\sinh\left(\alpha \sigma \right)\right]$ − 1/T.

The calculation process of material parameters in the Arrhenius model is illustrated with the example of a true strain of 0.2. Based on Equation (3), n_1_, β, and n values are obtained from the reciprocal of the slope of $ln\sigma -ln\dot{\varepsilon }$, $\sigma -ln\dot{\varepsilon },$ and $\ln\left[\sinh\left(\alpha \sigma \right)\right]$ − $ln\left(\dot{\varepsilon }\right)$ (Figure 4a–c); then, α = β/n_1_= 0.01623. Furthermore, the slope and intercept of $\ln\left[\sinh\left(\alpha \sigma \right)\right]$ − 1/*T* (Figure 4d) are the values of *Q*/nR and ($ln\left(\dot{\varepsilon }\right)-lnA)/n$; then, the values of *Q* and lnA are calculated as 179.4855 KJ/mol and 27.7726, respectively.

In addition, the Z parameter proposed by Zener and Hollomon can express the relationship between strain rate and deformation temperature, as follows [31]:

(4) $$Z=\dot{\varepsilon }\exp\left(\frac{Q}{RT}\right)=A{\left[sinh\left(\alpha \sigma \right)\right]}^{n}$$

Depending on the definition of hyperbolic law, the flow stress with the Z parameter can be given as:

(5) σ=1αlnZA1n+ZA2n+112

Therefore, the flow stress equation with a true strain of 0.2 can be established by substituting the values of material parameters α, n, and A into Equation (5) as:

(6) σ=61.614lnZ1.15×10120.2032+Z1.15×10120.4065+112

**Figure 5:**
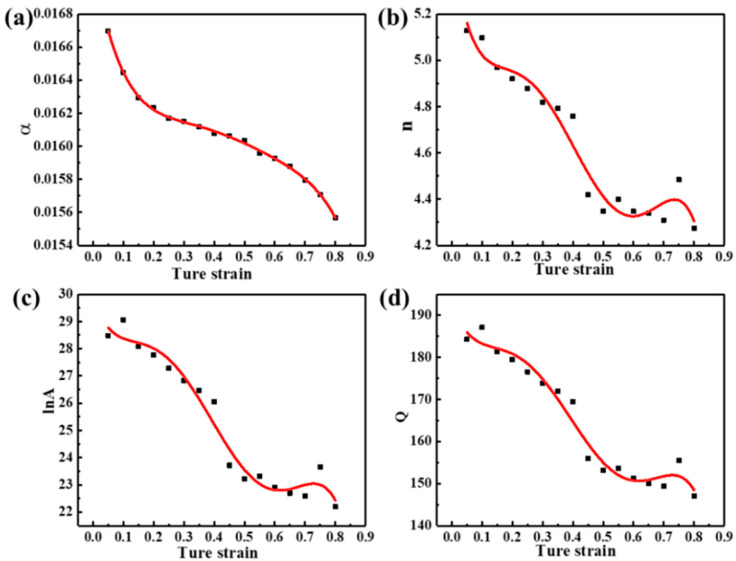
Fifth-order polynomial fitting curves of the material constants (**a**) α, (**b**) n, (**c**) lnA, and (**d**) Q to strain.

The above Arrhenius model does not consider the effect of strain on the flow behavior during hot deformation, merely considering the deformation temperature and the strain rate. To accurately describe the hot deformation behavior under different deformation conditions, an Arrhenius model considering strain compensation is required [5,32]. The material parameters (α, n, lnA, Q) in the constitutive equations can be calculated in a strain range of 0.05~0.8 (with an interval of 0.05). The functional relationship between each parameter and strain can be obtained by fitting a fifth-order polynomial, as shown in Equation (7). The fitting results are shown in Figure 5, and the corresponding polynomial coefficients are listed in Table 2.

(7) $$\left\{\begin{array}{c} \alpha =B_{0}+B_{1}\varepsilon +B_{2}{\varepsilon^{2}+B}_{3}\varepsilon^{3}{+B}_{4}\varepsilon^{4}+B_{5}\varepsilon^{5}\\ n=C_{0}+C_{1}\varepsilon +C_{2}{\varepsilon^{2}+C}_{3}\varepsilon^{3}{+C}_{4}\varepsilon^{4}+C_{5}\varepsilon^{5}\\ lnA=D_{0}+D_{1}\varepsilon +D_{2}{\varepsilon^{2}+D}_{3}\varepsilon^{3}{+D}_{4}\varepsilon^{4}+D_{5}\varepsilon^{5}\\ Q=E_{0}+E_{1}\varepsilon +E_{2}{\varepsilon^{2}+E}_{3}\varepsilon^{3}{+E}_{4}\varepsilon^{4}+E_{5}\varepsilon^{5} \end{array}\right.$$

**Figure 6:**
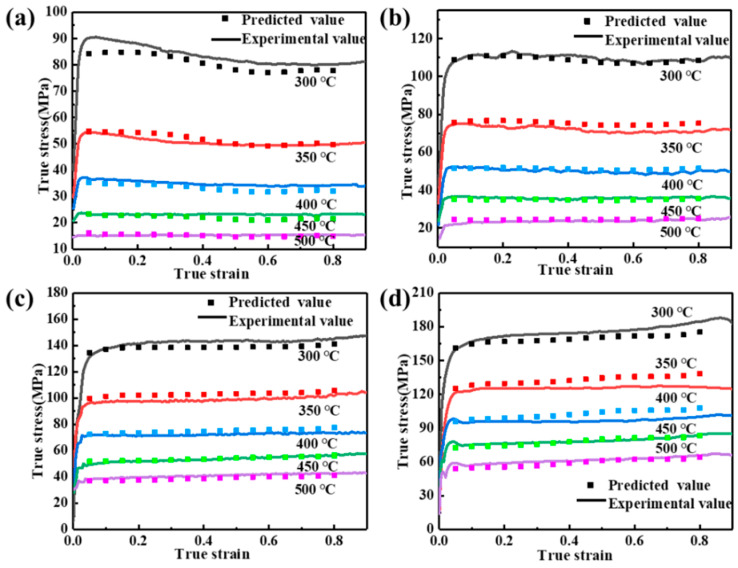
Comparison of the tested and predicted values of flow stress under different deformation conditions: (**a**) 0.001 s^−1^; (**b**) 0.01 s^−1^; (**c**) 0.1 s^−1^; (**d**) 1 s^−1^.

Hence, the flow stresses for different deformation conditions at any given strain can be predicted by the Arrhenius model, as shown in Equation (8). The predicted values of flow stress are compared with the experimental stress values, as presented in Figure 6.

(8) σ=1αεlnε˙exp⁡QεRTAε1nε+ε˙exp⁡QεRTAε2nε+112

**Figure 7:**
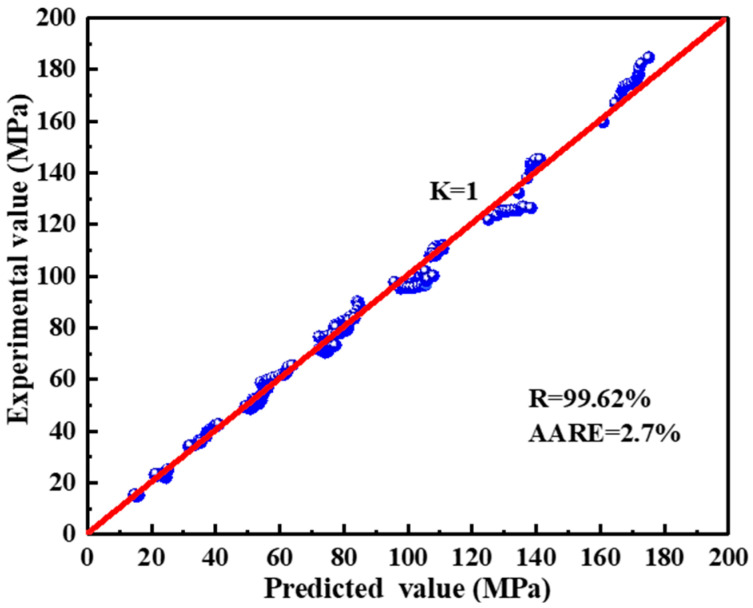
Correlations of the tested and predicted values of flow stress.

The prediction accuracy of the Arrhenius equation considering strain compensation can be indicated by the correlation coefficient (R) and the average absolute relative error (AARE), calculated from Equations (9) and (10), respectively. The calculated R = 99.62% is greater than 99% and the calculated AARE = 2.7% is less than 4%, which indicates that the constitutive model can better predict the flow stress under different deformation conditions [33,34]. Figure 7 shows the linear relationship between the tested and predicted values. Therefore, a constitutive equation can be used for the numerical simulation and hot deformation behavior analysis of 2050 Al-Cu-Li alloy.

(9) $$R=\frac{\sum_{i=1}^{N}\left(E_{i}-\bar{E}\right)\left(P_{i}-\bar{P}\right)}{\sqrt{\sum_{i=1}^{N}{\left(E_{i}-\bar{E}\right)}^{2}\sum_{i=1}^{N}{\left(P_{i}-\bar{P}\right)}^{2}}}$$

(10) $$AARE=\frac{1}{N}\sum_{i=1}^{N}\left|\frac{E_{i}-P_{i}}{E_{i}}\right|$$

### 3.3. Processing Map

To determine the most desirable hot deformation parameters, a hot-processing map was developed based on the dynamic material model (DMM) proposed by Prasad et al. [35]. The hot-processing diagram is produced by superimposing the power dissipation efficiency and instability diagrams. The power dissipation efficiency (*η*) denotes the proportion of the total power consumed by the evolution of the microstructure, as follows:

(11) $$\eta =\frac{2m}{m+1}$$

where *m* is the strain rate sensitivity index, as follows:

(12) $$m=\frac{\partial ln\sigma }{\partial ln\dot{\varepsilon }}$$

Generally, a greater value of *η* means a more sufficient level of dynamic softening during deformation and better hot workability. However, this is not absolute, because the value of η is high when flow instability (e.g., microcracks, adiabatic shear bands, and flow localization) occurs. Therefore, it is necessary to combine the instability criterion to prove the machinability of the alloy, as shown below [36]:

(13) $$\xi \left(\dot{\varepsilon }\right)=\frac{\partial ln\left[m/\left(m+1\right)\right]}{\partial ln\dot{\varepsilon }}+m$$

When $\xi \left(\dot{\varepsilon }\right)$ is less than 0, this indicates that flow instability will occur during the deformation process.

**Figure 8:**
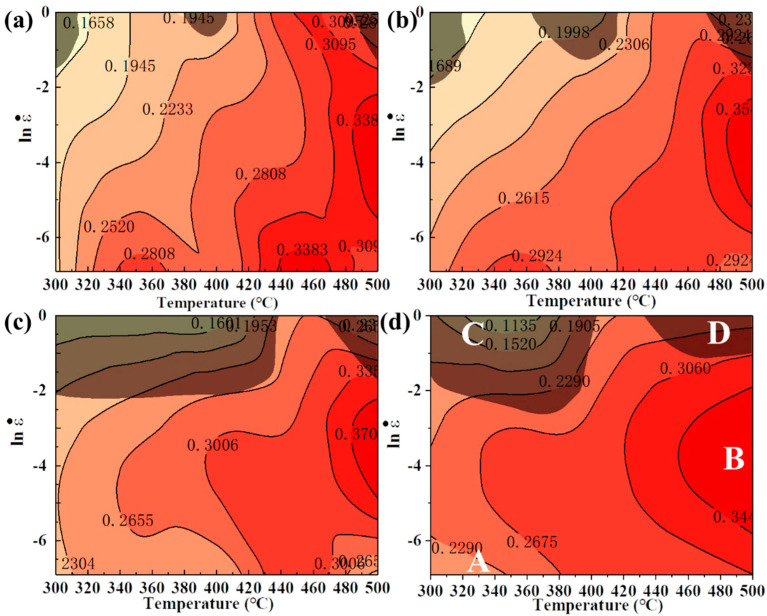
Processing maps at different strains: (**a**) ε = 0.2; (**b**) ε = 0.4; (**c**) ε = 0.6; (**d**) ε = 0.8. The grey-shaded areas indicate unstable areas and the remaining parts are safe areas. Areas A, B, C, and D in map (**d**) correspond to the deformation conditions of (300°C–380 °C, 0.1–0.001 s^−1^), (430°C–500 °C, 0.1–0.001 s^−1^), (300°C–400 °C, 0.1–1 s^−1^), and (440°C–500 °C, 0.1–1 s^−1^), respectively.

The hot-processing maps for different strains are shown in Figure 8. The values on the contour are the values of η, and the gray area is the instability region where the parameter $\xi \left(\dot{\varepsilon }\right)$ is less than 0. For true strains of 0.2, 0.4, 0.6, and 0.8, power dissipation efficiency η varies similarly for different deformation temperatures and strain rates. There is a noticeable effect of deformation temperature on the power dissipation factor, and the value of η increases with increasing deformation temperature; however, the strain rate effect on the value of η is only significant at low temperatures. The lowest η values occur in the low-temperature and high-strain-rate regions, and the highest η values are found in the high-temperature and medium-strain-rate regions. However, there is no maximum at high temperatures and in low-strain-rate conditions since the slow strain rates provide enough time for the occurrence of DRV, thus reducing the DRX driving force [5]. Theoretically, the dissipation value caused by DRX is larger than that caused by DRV, owing to more stored energy being required for DRX grain nucleation and grain boundary migration [9]. Previous research showed that efficiency values of 20~30% correspond to DRV and 35~45% correspond to DRX [14]. In addition, the region of flow instability varies with increasing strain. At true strains of 0.8, the destabilization region is mainly concentrated in the low- or medium-temperature and high strain-rate region (300 °C–400 °C, 0.1–1 s^−1^, marked with zone C) and the high-temperature and high strain-rate region (440 °C–500 °C, 0.1–1 s^−1^, marked with zone D). Furthermore, the power dissipation values are higher in zone B (430 °C–500 °C, 0.1–0.001 s^−1^) and lowest in zone A (300 °C–380 °C, 0.1–0.001 s^−1^) in the safety zone.

### 3.4. Microstructure Characterization

**Figure 9:**
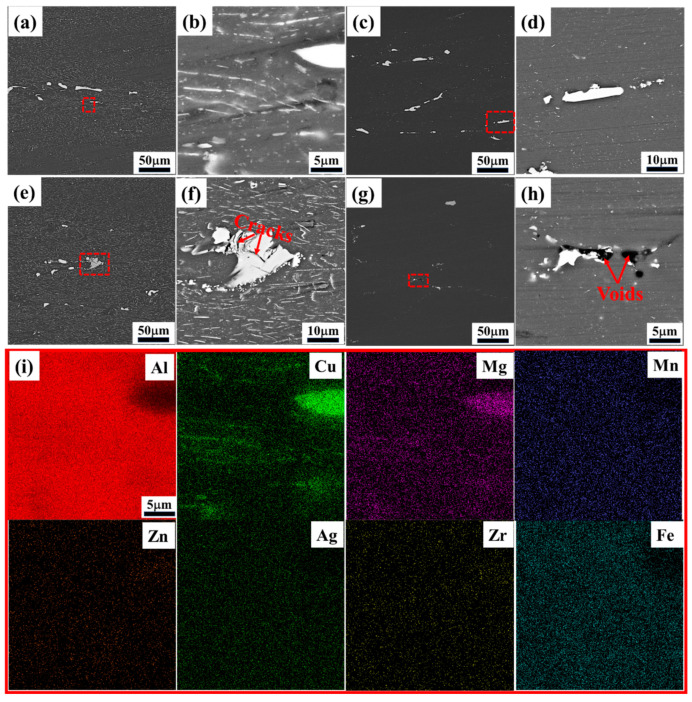
SEM images of specimens subjected to hot compression deformation under the following conditions: (**a**,**b**) 300 °C/0.001 s^−1^; (**c**,**d**) 500 °C/0.01 s^−1^; (**e**,**f**) 350 °C/1 s^−1^; (**g**,**h**) 500 °C/1 s^−1^; and (**i**) EDS image corresponding to (**b**).

It is necessary to analyze the microstructure of the machinable region and the flow instability region to verify the reliability of the hot-processing map. Plastic instability is associated with the coarsening and de-bonding or cracking of the secondary phase, in addition to flow localization and adiabatic shear bands in the microstructure [37]. Therefore, the evolution of the secondary phase under different deformation conditions has a significant influence on hot deformation behavior. SEM images of the four specimens in regions A, B, C, and D of the hot-processing map (Figure 8) are given in Figure 9, respectively. Under both high- and low-temperature deformation conditions, there are coarse secondary phases in the α-Al crystals, which originate mainly from the undissolved primary phases occurring during the homogenization process. In addition, a large number of finely dispersed secondary phases can be observed in the high-magnification SEM images (Figure 9b,d,f,g) of the specimens deformed at low temperatures of 300 °C–350 °C, whereas there are fewer at high temperatures of 500 °C, which finding is related to the exsolution of the highly saturated solid solution under low-temperature conditions. Normally, precipitation does not occur in alloys heat-treated at the same deformation temperature but that have not deformed; however, defects (e.g., vacancies, dislocations, and stacking faults) that are introduced during deformation improve the solute atomic activity and promote diffusion, which facilitates dynamic precipitation [38]. Dynamic precipitation phenomena occurring during deformation under low-temperature conditions have been reported in numerous studies, and the introduction of defects increased the nucleation rate of DPN by 10^590^ times [7,39,40]. The EDS results from Figure 9i show that Cu and Mg atoms are heavily aggregated on both the coarse primary phase and the fine precipitates, while the other elements are more evenly distributed, suggesting that they are Al-Cu/Al-Cu-Li phases enriched with Mg elements. For third-generation Al-Cu-Li alloys, the dynamic precipitation that occurs during hot deformation at low or medium temperatures is mainly dominated by the Al_2_CuLi phase, as demonstrated in numerous studies [41,42,43]. Based on the morphological characteristics of the precipitates and our previous study [38], it can be asserted that the needle-like phase that undergoes bending or twisting is the Al_2_CuLi phase.

Moreover, cracks and micro-voids are evident on and near the coarse primary phase in Figure 9f,h, respectively. Under the conditions of 350 °C/1 s^−1^, meanwhile, with the dynamic precipitation of secondary phase pinning dislocations, inhibiting the dynamic softening that occurs due to dislocation annihilation or rearrangement, the strain-strengthening effect is increased, and then the greatest stress focuses on the coarse primary phase [44]. When the stress is greater than the limit of the primary phase, the primary phase breaks and releases energy, leading to flow instability. For a specimen deformed at 500 °C/1 s^−1^, the activatable slip system number increases at high temperatures, and the plastic deformation capacity of the Al alloy improves. However, this then increases the gap in deformation ability between α-Al crystals and coarse particles, greatly weakening the interfacial relationship between α-Al crystals and coarse particles due to the different coefficients of hot expansion [6,45]. Therefore, the stresses generated by the lattice distortion occurring in the Al cannot be perfectly transferred to the hard particles at high strain rates, and debonding is easily induced at their interfaces; this forms micro-voids and contributes to the development of flow instability [46].

**Figure 10:**
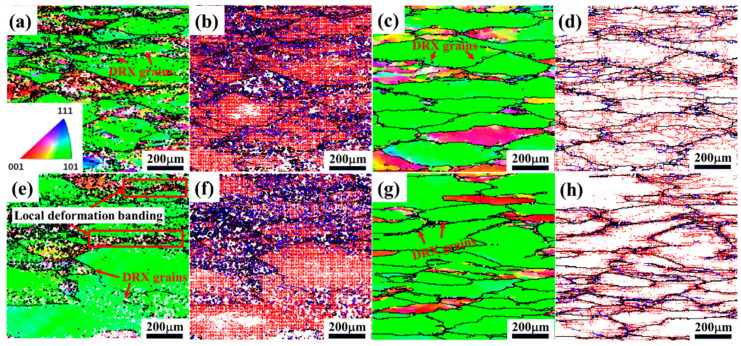
The inverse pole figure (IPF) and grain boundary (GB) maps for deformation conditions at (**a**,**b**) 300 °C/0.001 s^−1^, (**c**,**d**) 500 °C/0.01 s^−1^, (**e**,**f**) 350 °C/1 s^−1^, and (**g**,**h**) 500 °C/1 s^−1^.

As shown in Figure 10, EBSD characterization analyses were carried out for samples with deformation conditions of 300 °C/0.001 s^−1^ and 500 °C/0.01 s^−1^ in the safe zones of A and B (Figure 8d) and 350 °C/1 s^−1^ and 500 °C/1 s^−1^ in the unstable flow zones of C and D, respectively. The microstructure is mainly composed of numerous deformed grains elongated along the direction vertical to the compression direction and also some fine equiaxed DRX grains. Different colors represent different orientations in the IPF images, and it can be seen that the majority of deformed grains exhibit the <101> orientation. Hu et al. [47] pointed out that for Al alloys with an FCC structure, most of the grains rotate in a certain direction during compression; this direction is called “stable-end orientation”. Hence, it indicates that the stable-end orientation of the 2050 Al-Cu-Li alloy compression is the [101]//CD (CD: compression direction) fiber; similar phenomena were reported by Tiamiyu et al. [48] with AA 2624 alloy. At the low deformation temperature shown in Figure 10a,e, there are plenty of fine grains with different orientations appearing inside some of the deformed grains in the IPF diagrams. Moreover, it is evident that a significant number of zero-resolution points are present, compared to those samples under high-temperature deformation. These are associated with the massive dynamic precipitation of fine secondary phases as a result of low-temperature deformation, as evidenced by the above SEM results in Figure 9. The precipitation of the secondary phase during deformation can effectively promote the development of DRX through the PSN mechanism; a similar phenomenon has been identified in some reports during the hot deformation of Al alloy [49,50]. Compared to the sample at 350 °C/1 s^−1^ shown in Figure 10e, the deformation microstructure is homogeneously oriented at 300 °C/0.001 s^−1^, 500 °C/0.01 s^−1^, and 500 °C/1 s^−1^, which suggests that the alloys are better processed plastically at high temperatures or under low strain rates. Furthermore, the local deformation band is observed in the sample under deformation conditions of 350 °C/1 s^−1^. Under low-temperature and high strain-rate conditions, higher resistance to plastic deformation exists in the alloy, while the heat released from deformation cannot be transferred quickly; consequently, rapidly rising local temperature causes deformation to occur violently [51].

**Figure 11:**
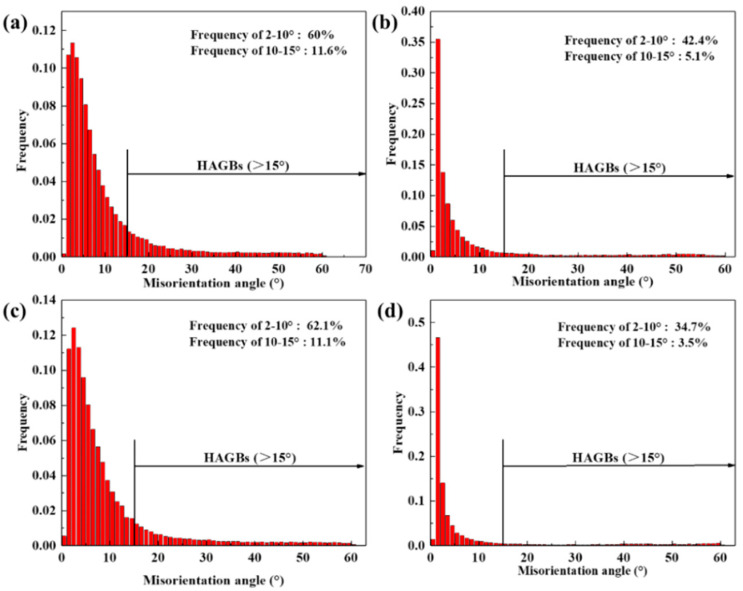
GB misorientation angle distribution maps under (**a**) 300 °C/0.001 s^−1^, (**b**) 500 °C/0.01 s^−1^, (**c**) 350 °C/1 s^−1^, and (**d**) 500 °C/1 s^−1^.

The black lines in the grain boundary (GB) distribution diagram (Figure 10b,d,f,h) denote high-angle grain boundaries (GB misorientation angle > 15°, HAGBs), the blue lines denote medium-low-angle grain boundaries (GB misorientation angle of 10–15°, MLGBs), and the red lines denote low-angle grain boundaries (GB misorientation angle of 2–10°, LAGBs). It is clear that both LAGBs and MLGBs are significantly higher at the low temperatures in Figure 10b,f than at the high temperatures in Figure 10d,h, and the statistical distribution of GB misorientation is shown in Figure 11. The low hot activation energy provided at low temperatures, combined with the effective pinning of dislocations and (sub)grain boundaries by abundant dynamically precipitated fine secondary phases, results in the entanglement and stacking of extensive dislocations. These sufficient amounts of LAGBs continuously absorb these dislocations and transform into MLGBs.

**Figure 12:**
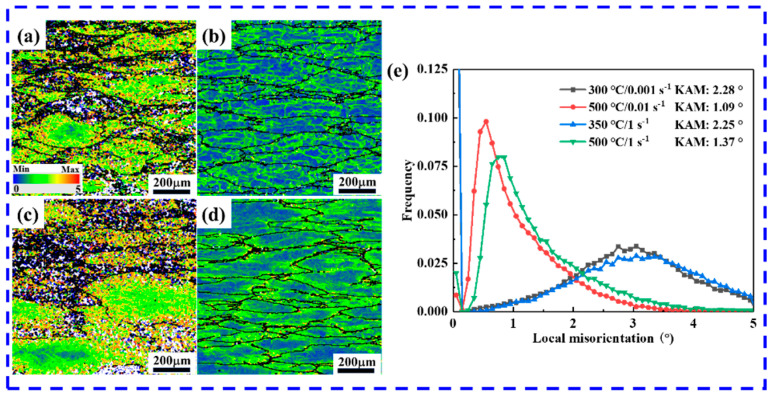
KAM maps of samples at (**a**) 300 °C/0.001 s^−1^, (**b**) 500 °C/0.01 s^−1^, (**c**) 350 °C/1 s^−1^, and (**d**) 500 °C/1 s^−1^; (**e**) the corresponding variations in KAM values. KAM map, with the blue color indicating the lowest dislocation density/strain regions and the red color indicating the highest dislocation density/strain regions.

The kernel average misorientation (KAM) value is positively correlated with the geometrically necessary dislocation (GND), responding effectively to the variations in dislocation density [52]. To characterize the dislocation density and stress distribution, KAM maps for the different deformation conditions are given in Figure 12a–d. Since both dislocations and slip bands terminate near the grain boundaries, leading to blockage and, thus, stress concentration, the KAM values near the deformed grain boundaries are significantly larger than those within the grain boundaries, especially at high temperatures and with low or high strain rates, as shown in Figure 12b,d. Compared to the high-temperature figures shown in Figure 12b,d, significantly more orange areas appear in Figure 12a,c at low temperatures, indicating higher dislocation density and, hence, greater stress, which is consistent with the analysis of the stress–strain curves shown in Figure 3. The average KAM values for different deformation conditions are shown in Figure 12e, and the results demonstrate that the KAM values keep decreasing with the increase in temperature and decrease in the strain rate. Compared to the strain rate, the deformation temperature has a greater effect on KAM values. It can be seen from Figure 12e that the KAM values at high temperatures are mainly concentrated in the smaller range of 0–2; at low temperatures, they are mainly concentrated in the range of 2–4, while there is not much difference in the distribution of KAM values under high and low strains. Generally, the higher thermal activation energy provided by the high-temperature deformation conditions is more favorable for dislocation annihilation and rearrangement, and DRV and DRX occur more efficiently, indicating the presence of more strain-free zones, and, hence, a higher fraction of low KAM values [53]. Conversely, the greater number of dislocations and substructures seen during low-temperature deformation results in increased stored energy and greater KAM values.

**Figure 13:**
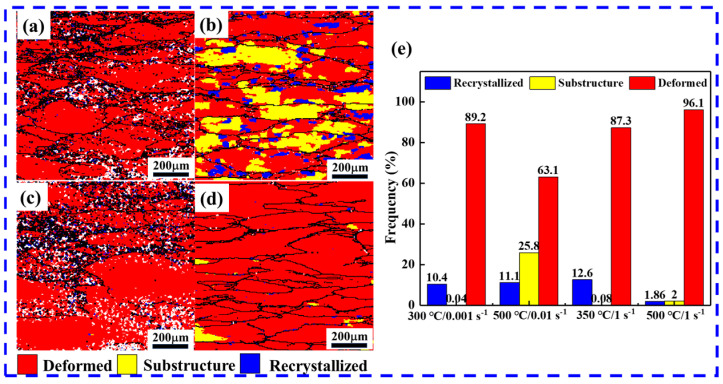
Deformation microstructure distribution maps of samples at (**a**) 300 °C/0.001 s^−1^, (**b**) 500 °C/0.01 s^−1^, (**c**) 350 °C/1 s^−1^, and (**d**) 500 °C/1 s^−1^; (**e**) the deformation microstructure fraction.

Figure 13 shows the distribution of deformed, sub-structured, and recrystallized samples under different deformation conditions, where the blue color represents the DRX grain, the yellow indicates the substructure, and the red represents the deformed structure. Some DRX grains are observed in Figure 13a–c, with fewer in Figure 13d. The appearance of DRX grains at low temperatures is related to the PSN mechanism induced by precipitates (Figure 13a,c). In Figure 13b, the deformation, substructure, and recrystallized tissue are found at high temperatures and low strain rates. DRX grains at low temperatures are significantly smaller than DRX grains at high temperatures, which is due to the increased ability regarding dislocations and (sub)grain boundary movement and the absence of the precipitated fine secondary phases at high temperatures. Several studies have reported that the development of DRX during hot deformation is supported by high temperatures and low strain rates [5,54]. However, in this paper, it was surprising to notice that recrystallization occurs at low temperatures; the results are contrary to those of most reports, in which they are mainly attributed to the promotion of DRX by dynamic precipitation during the deformation of a highly saturated solid solution of Al alloy at low temperatures. The statistics of the percentage of microstructures under different deformation conditions are shown in Figure 13e. At deformations of 300 °C/0.001 s^−1^ and 350 °C/1 s^−1^, the deformed structures are 89.2% and 87.3%, and the recrystallized structures are 10.4% and 12.6%, respectively.

**Figure 14:**
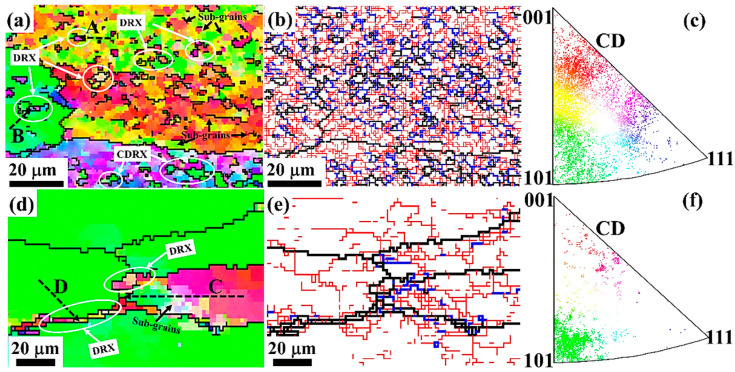
EBSD analysis at high magnification of samples at (**a**–**c**) 300 °C/0.001 s^−1^ and (**d**–**f**) 500 °C/0.01 s^−1^: (**a**,**d**) IPF maps; (**b**,**e**) GB maps; (**c**,**f**) scattered IPF.

**Figure 15:**
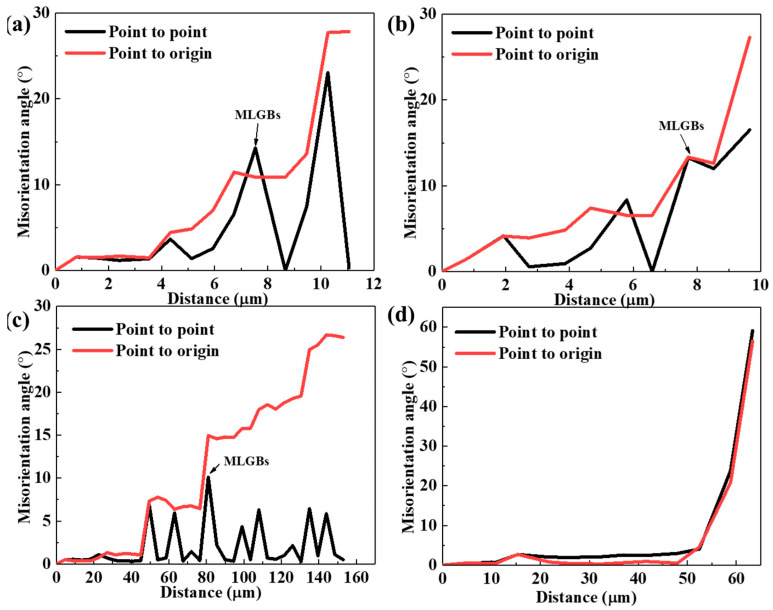
Variation in misorientation angles along (**a**) line A, (**b**) line B, (**c**) line C, (**d**) line D in Figure 14a,d.

To investigate the DRX mechanism of the samples with deformation conditions at 300 °C/0.001 s^−1^ and 500 °C/0.01 s^−1^ in the safe area, the enlarged IPF maps, corresponding GB distributions, and scattered IPF in Figure 14 were analyzed. In the sample with the deformation conditions of 300 °C/0.01 s^−1^, numerous fine DRX grains promoted by the PSN mechanism were generated inside the deformed grains, with the same distribution as that of the CDRX grains. The misorientation variations of the A and B lines in Figure 14a from the deformed grain to DRX grains are shown in Figure 15a,b. The misorientation (point to origin) shows a monotonically increasing trend for both line A and line B, suggesting that the DRX grains were obtained by the progressive rotation evolution of sub-grains, which is consistent with the mechanism of CDRX. In Figure 14d,e, DRX grains and some irregularly shaped closed and semi-closed sub-grains with different orientations encircled by LAGBs and MLGBs are gathered near the grain boundaries in samples with deformation conditions of 500 °C/0.01 s^−1^. The movement of dislocations generated by deformation was obstructed and then clustered around the grain boundaries. As a result of the annihilation and rearrangement of dislocations, sub-grains with multiple orientations were formed [48]. To further reduce the energy, LAGBs absorbed the dislocations and transformed into MLGBs. The variation in “point to origin” misorientation and the presence of MLGBs in “point to point” misorientation along C in Figure 15c indicates that the DRX mechanism is CDRX. MLGBs have been reported to be characteristic of CDRX grains obtained by sub-grain rotation [16]. Moreover, a sharp increase to 15° in the misorientation of “point to origin” is found along line D in Figure 15d, and the grain morphology is of a necklace-like shape, which is typical of the DDRX mechanism.

## 4. Conclusions

For this article, the isothermal hot compression tests of homogenized 2050 Al-Cu-Li alloy were carried out at deformation temperatures of 300 °C–500 °C and strain rates of 0.001 s^−1^–1 s^−1^. An Arrhenius-type model that accurately predicts the flow stresses with different deformation conditions and a hot-processing map that predicts the workable parameters were established. Combined with the true stress–strain curve and microstructure characterization, the hot deformation behavior and the deformation mechanism in different processing regions were revealed, and the main conclusions are as follows:

1. The flow stress of 2050 Al-Li alloy increases significantly with true strain (true strain of less than 0.05) in the early compression process and then stabilizes. The highest flow stresses occur at high strain rates and low temperatures, and the flow stress for various deformation conditions can be accurately described by the established strain-compensated Arrhenius model.
2. The appropriate conditions for the hot working of 2050 Al-Li alloys have been identified as 430 °C–500 °C and 0.1 s^−1^–0.001 s^−1^, where the dynamic softening mechanisms are dynamic recovery (DRV) and dynamic recrystallization (DRX), and the DRX mechanisms are dominated by DDRX and CDRX, which exhibit a large dissipation efficiency (> 30%).
3. The processing parameter regions C (300 °C–400 °C, 0.1–1 s^−1^) and D (440 °C–500 °C, 0.1–1 s^−1^) are undesirable for 2050 Al-Li alloys, due to their inhomogeneous microstructure and the occurrence of flow instability, in which local deformation banding and secondary phase cracking are mainly observed in region C, while micro-voids are formed in region D.
4. The microstructure of the compressed specimens consists of abundant pancake-like grains with an orientation concentrated in the <101>CD direction, along with some equiaxed DRX grains with a random orientation. Compared to samples under high-temperature conditions, a considerable population of DRX grains with smaller sizes was observed at low temperatures, accompanied by a higher number of LAGBs and HAGBs, which finding is related to the PSN mechanism induced by dynamic precipitation.

## Figures and Tables

**Table 1:** Chemical composition of 2050 Al-Cu-Li alloy (wt. %).

Cu	Li	Mg	Mn	Ag	Zn	Zr	Fe	Al
3.30	0.77	0.54	0.31	0.26	0.19	0.13	0.01	Bal

**Table 2:** Coefficients of the fifth polynomial fitting of the material constants α, n, lnA, and Q.

	α		n		lnA		Q
B0	0.0171	C0	5.48541	D0	29.86473	E0	192.91543
B1	−0.01008	C1	−9.17561	D1	−32.47934	E1	−203.7002
B2	0.04411	C2	63.24509	D2	260.26328	E2	1572.6177
B3	−0.09778	C3	−203.4514	D3	−979.0298	E3	−5796.022
B4	0.10441	C4	276.17689	D4	1431.5919	E4	8405.2129
B5	−0.04396	C5	−132.0563	D5	−711.4815	E5	−4160.178

## Data Availability

No new data were created or analyzed in this study.
